# Artificial double inversion recovery images can substitute conventionally acquired images: an MRI-histology study

**DOI:** 10.1038/s41598-022-06546-4

**Published:** 2022-02-16

**Authors:** Piet M. Bouman, Martijn D. Steenwijk, Jeroen J. G. Geurts, Laura E. Jonkman

**Affiliations:** Department of Anatomy and Neurosciences, MS Center Amsterdam, Amsterdam Neurosciences, Amsterdam UMC, Vrije Universiteit Amsterdam, De Boelelaan 1117, 1081 HV Amsterdam, The Netherlands

**Keywords:** Neuroscience, Medical research, Neurology, Signs and symptoms

## Abstract

Cortical multiple sclerosis lesions are disease-specific, yet inconspicuous on magnetic resonance images (MRI). Double inversion recovery (DIR) images are sensitive, but often unavailable in clinical routine and clinical trials. Artificially generated images can mitigate this issue, but lack histopathological validation. In this work, artificial DIR images were generated from postmortem 3D-T1 and proton-density (PD)/T2 or 3D-T1 and 3D fluid-inversion recovery (FLAIR) images, using a generative adversarial network. All sequences were scored for cortical lesions, blinded to histopathology. Subsequently, tissue samples were stained for proteolipid protein (myelin) and scored for cortical lesions type I-IV (leukocortical, intracortical, subpial and cortex-spanning, respectively). Histopathological scorings were then (unblinded) compared to MRI using linear mixed models. Images from 38 patients (26 female, mean age 64.3 ± 10.7) were included. A total of 142 cortical lesions were detected, predominantly subpial. Histopathology-blinded/unblinded sensitivity was 13.4/35.2% for artificial DIR generated from T1-PD/T2, 14.1/41.5% for artificial DIR from T1-FLAIR, 17.6/49.3% for conventional DIR and 10.6/34.5% for 3D-T1. When blinded to histopathology, there were no differences; with histopathological feedback at hand, conventional DIR and artificial DIR from T1-FLAIR outperformed the other sequences. Differences between histopathology-blinded/unblinded sensitivity could be minified through adjustment of the scoring criteria. In conclusion, artificial DIR images, particularly generated from T1-FLAIR could potentially substitute conventional DIR images when these are unavailable.

## Introduction

Cortical grey matter has a profound role in the pathophysiology of multiple sclerosis, being present in early stages of the disease and being associated with disease progression and conversion^1–4^. Cortical lesions are highly disease specific, and hence included in the multiple sclerosis diagnostic criteria^5^. Nevertheless, cortical lesions are inconspicuous on MRI: histopathological sensitivity of conventional clinical sequences (i.e., T_1_, T_2_, and fluid-attenuated inversion recovery (FLAIR)) lies beneath 10%^6–8^. Double inversion recovery (DIR) has been found to be more sensitive (i.e., 18–23%), and highly pathologically specific (i.e., > 90%). With DIR imaging, signals from both the cerebrospinal fluid and the white matter are selectively suppressed, providing an image in which the grey matter is emphasized^9^. However, DIR sequences are not pre-programmed on all MR systems and are time-consuming in their acquisition. Consequently, they are not readily available in all hospitals and are omitted in most clinical trials.

This issue has recently been mitigated by the introduction of artificially generated DIR images^10,11^. This technology, that has been used in different settings in medical imaging before^12,13^, aids the availability of DIR in the clinical settings and enables ex post facto implementation of DIR in e.g. clinical trials cohorts. Aided by convolutional neural networks, DIR images can be generated from a combination of other –e.g. conventional– sequences, such as T_1_-weighted and PD/T_2_ or T_1_-weighted and FLAIR. Artificially generated DIR images were found to detect an equal number of cortical lesions compared to conventionally acquired DIR images; however, histopathological validation is lacking to date^10,11^.

The objective of the current work was to compare the sensitivity and specificity of artificially generated DIR images for cortical lesion detection in patients with multiple sclerosis, set against conventionally acquired DIR images and conventional (‘standard’) 3D-T_1_-weighted images, using histopathology as gold standard. Thereby, we aim to assess whether artificially generated DIR images could potentially supplement conventional clinical images in hospitals and clinical trials.

## Results

Following in situ MRI and tissue availability, MRI data from thirty-eight patients (mean age 63.3 years, standard deviation (SD) 10.7 years; 31% male; mean postmortem delay at commencement of autopsy protocol 3:55 h SD 0:57 h) with progressive multiple sclerosis (mean disease duration 26.6 years, SD 10.9 years; 25 secondary progressive, 8 primary progressive, 5 unknown) were included for cortical lesion scoring. Thirteen patients were scanned using a 1.5 T Siemens Sonata system, twenty-five patients were scanned using a 3 T GE Signa HDxt system. From twenty-three patients, tissue samples were available. An overview of patient details is provided in Table 1. Overviews of the imaging training results (i.e., input and output) are provided in Figures S1 and S2. A total of 142 cortical lesions were identified in 66 tissue samples. Of these lesions, 6 were type I, 47 were type II, 76 were type III and 13 were type IV. In addition, 7 white matter lesions were detected.

**Table 1:** Demographic and clinical measures.

	Post-mortem (n = 38)
Female, *n* (%)	26 (69)
Age, years	64.3 (10.7)
PMD (h:mm)	3:55 (0:57)
DD, years	26.6 (10.9)
Disease type	25 SPMS, 8 PPMS, 5 unknown
EDSS^a^	10.0 (0)

Description of demographic and clinical characteristics for the post-mortem (left) and in-vivo (right) cohorts. All values represent means and SD, unless otherwise denoted. PMD = post-mortem delay, SPMS = secondary progressive multiple sclerosis, PPMS = primary progressive multiple sclerosis, RRMS = relapsing–remitting multiple sclerosis, EDSS = Expanded Disability Status Scale.

^a^Median and range.

### Histopathology-blinded lesion detection

**Figure 1:**
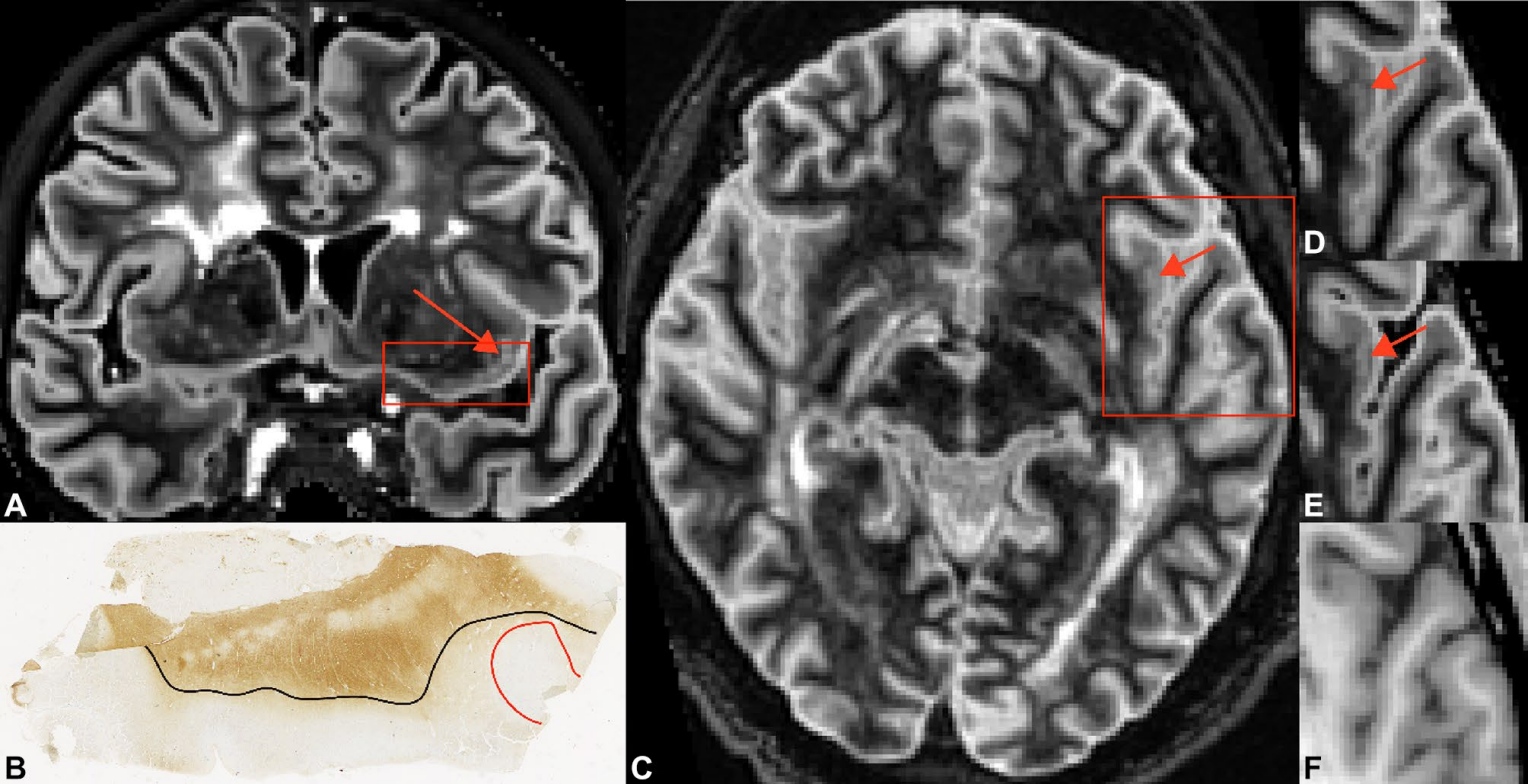
Example of a type IV cortical lesion in the insular cortex, that is a priori visible on both artificially generated and conventionally acquired DIR images but not on standard 3D-T_1_. (**A**) Coronal slice in which the histopathological sample is indicated by the rectangle; the arrow indicates a visible type IV lesion. (**B**) Histopathological tissue sample with the type IV lesion indicated by the red line and the cortex by the black line. (**C**) Conventionally acquired DIR image with the type IV lesion indicated by the arrow; the rectangle demarcates the inset that is being shown in images D-F. (**D**) Inset of the corresponding artificial DIR image generated from 3D-T_1_ and 3D-FLAIR with the type IV lesion indicated by the arrow. (**E**) Inset of the corresponding artificial DIR image generated from 3D-T_1_ and PD/T_2_, with the type IV lesion indicated by the arrow. (**F**) Inset of the corresponding standard 3D-T_1_ image, in which the type IV lesion is not visible (generated using Adobe Illustrator—Adobe Inc., 2019. Adobe Illustrator, Available at https://adobe.com/products/illustrator).

The number of detected cortical lesions and the sensitivity measures corresponding to those numbers are displayed in Table 2. No statistical differences were found between conventional DIR sequences and artificial DIR sequences (both generated from T_1_-PD/T_2_ and T_1_-FLAIR) and 3D-T_1_ in the histopathology-blinded scoring. Descriptively, conventional DIR detected few more cortical lesions than T_1_-PD/T_2_ generated DIR, with 25 versus 19 cortical lesions, and also few more cortical lesions than T_1_-FLAIR generated DIR, with 25 versus 20 cortical lesions. The largest differences were found in type III lesions, of which 12 were detected on conventional DIR and 3 on 3D-T_1_. In Fig. 1, an example of a cortical lesion that was scored blinded to histopathology is depicted. The difference between conventional DIR and 3D-T_1_ closely approached, but fell short of significance (*P* = 0.07).

**Table 2:** Lesion count (sensitivity in %) MRI scoring.

Histology		MRI rating							
Lesion type	N	Blinded	Unblinded	Blinded	Unblinded	Blinded	Unblinded	Blinded	Unblinded
		Conventional DIR	Conventional DIR retro	DIR from 3D-T_1_ + PD/T_2_ pro	DIR from 3D-T_1_ + PD/T_2_ retro	DIR from 3D-T_1_ + FLAIR pro	DIR from 3D-T_1_ + FLAIR retro	3D-T_1_ pro	3D-T_1_ retro
Type I	6	5 (83.3)	5 (83.3)	4 (75.0)	5 (83.3)	5 (83.3)	5 (83.3)	4 (75.0)	5 (83.3)
Type II	47	0 (0.0)	3 (6.4)	0 (0)	2 (4.3)	0 (0.0)	2 (4.3)	0 (0.0)	3 (6.4)
Type III	76	12 (15.8)	51 (67.1)	6 (7.9)	32 (42.1)	7 (9.2)	42 (55.3)	3 (3.9)	31 (40.8)
Type IV	13	8 (61.5)	11 (84.6)	9 (69.2)	11 (84.6)	8 (61.5)	10 (76.9)	8 (61.5)	10 (76.9)
Type I-IV	142	25 (17.6)	70 (49.3)	19 (13.4)	50 (35.2)*	20 (14.1)	59 (41.5)	15 (10.6)	49 (34.5)*
WML	7	6 (85.7)	6 (85.7)	7 (100)	7 (100)	6 (85.7)	6 (85.7)	7 (100)	7 (100)
Total	149	31	76	26	57	26	65	22	46

N = number of detected/discernible cortical lesions; WML = white matter lesion.

*Significant differences (i.e. *P* < 0.05) compared to conventional DIR.

Histopathology-blinded intra-rater intraclass correlation coefficients were 0.986 for conventional DIR, 0.974 for T_1_-PD/T_2_ generated DIR, 0.997 for T_1_-FLAIR-generated DIR, and 0.979 for 3D-T_1_. Histopathology-blinded inter-rater intraclass correlation coefficients were 0.951 for conventional DIR, 0.946 for T_1_-PD/T_2_ generated DIR, 0.941 for T_1_-FLAIR generated DIR and 0.800 for 3D-T_1_.

**Figure 2:**
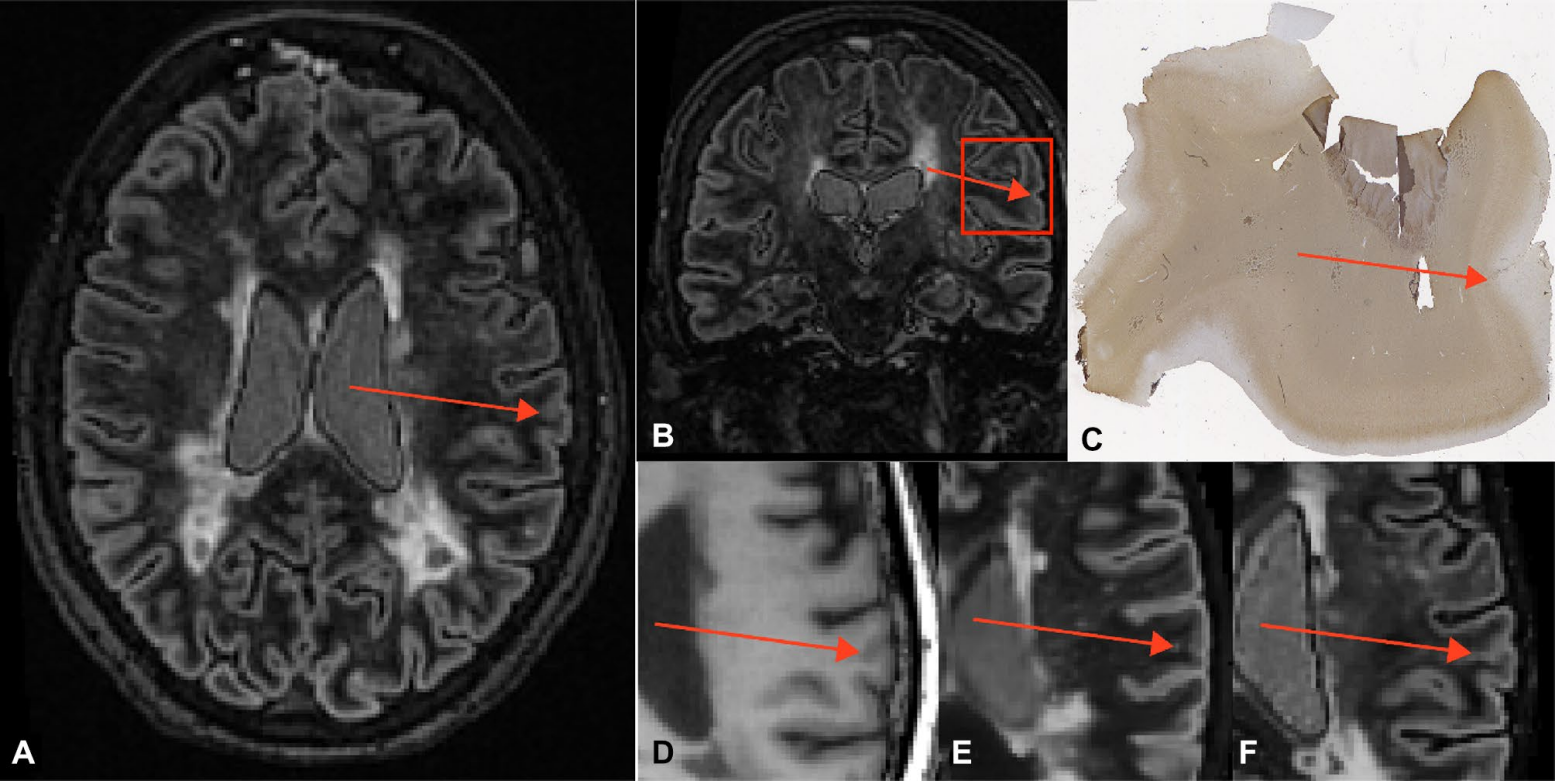
False-positive. Example of a ‘false-positive’, an area of partial demyelination or remyelination that has been scored as a cortical lesion on all (conventional and artificial) DIR sequences. The panels depict the conventionally acquired DIR image in axial fashion (**A**), a coronal image as a reference for the histopathological sample (**B**), the histopathological sample that has been stained for myelin (**C**), 3D-T_1_ (**D**), artificial DIR generated from 3D-T_1_ and PD/T_2_ (**E**), and artificial DIR generated from 3D-T_1_ and FLAIR (**F**). The partial demyelination/remyelination area is indicated by the red arrow on all panels (generated using Adobe Illustrator—Adobe Inc., 2019. Adobe Illustrator, Available at https://adobe.com/products/illustrator).

A total of eight false positives (areas that were scored as cortical lesions but did not appear to be lesions upon histopathological validation) were found in the data: 1 out of 25 on conventional DIR (4.0%), 2 out of 19 on T_1_-PD/T_2_ generated DIR (10.5%), 2 out of 20 on T_1_-FLAIR generated DIR (10.0%) and 3 out of 15 on 3D-T_1_ (20.0%). Specificity was 96.0% for conventional DIR, 89.5% for T_1_-PD/T_2_ generated DIR, 90.0% for T_1_-FLAIR generated DIR, and 80.0% for 3D-T_1_. An example of a partially demyelinated/remyelinating area that was scored as cortical lesion on both artificially generated and conventionally acquired DIR images is shown in Fig. 2.

### Lesion discernibility unblinded to histopathology

**Figure 3:**
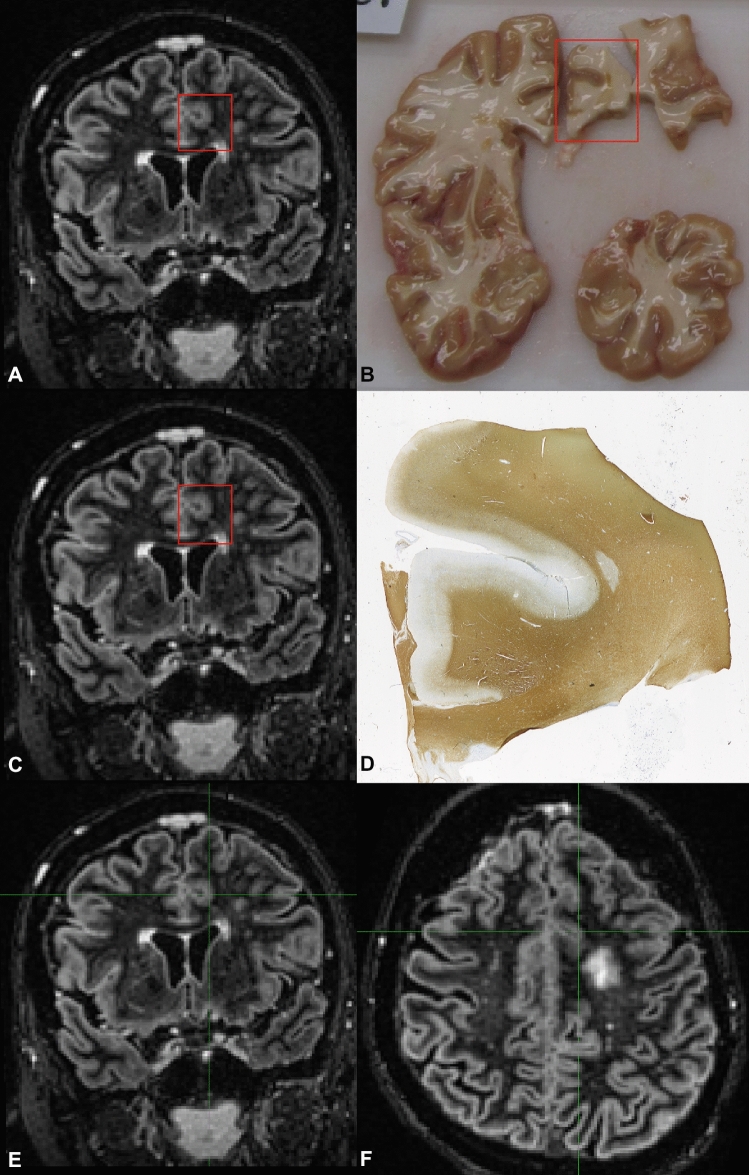
Overview of the histopathological tissue sample to MRI matching procedure. (**A**) The coronal double inversion recovery (DIR) MRI is matched to (**B**) a photograph of the tissue sample. Then, the coronal DIR MRI (**C**) is matched to the histopathological sample (**D**) coming from the tissue sample. Next, the cross-hair is used to translate the histopathological tissue sample location from the coronal DIR (**E**) to the axial DIR (**F**); (generated using Adobe Illustrator—Adobe Inc., 2019. Adobe Illustrator, Available at https://adobe.com/products/illustrator).

Prior to commencing the unblinded scoring, the reproducibility of MRI-histopathology matching (as displayed in Fig. 3) was evaluated by repeating the manual identification of MRI regions for 34% (n = 27 samples) of the data, and showed a reproducibility of 92.6%. In the unblinded scoring, i.e. direct comparison of the tissue samples matched to MRI, an increase in detection was found for all sequences. Conventionally acquired DIR outperformed artificial DIR from T_1_ and PD/T_2_ (*P* = 0.02) and 3D-T_1_ (*P* = 0.01) with 70 vs. 50 and 49 lesions, respectively. These differences were predominantly driven by type III lesions. With histopathological feedback available, there were no differences between conventionally acquired DIR and artificial DIR from T_1_ and FLAIR, nor between the other sequences. There were no differences in terms of specificity of the images that were generated from 1.5 T and 3 T data.

Unblinded intra-rater intraclass correlation coefficients were 0.929 for conventional DIR, 0.960 for T_1_-FLAIR generated DIR, 0.940 for T_1_-PD/T_2_ generated DIR, and 0.710 for 3D-T_1_. Unblinded inter-rater intraclass correlation coefficients were 0.909 for conventional DIR, 0.800 for T_1_-PD/T_2_ generated DIR, 0.750 for T_1_-FLAIR generated DIR and 0.833 for 3D-T_1_.

### Image contrasts

Contrast ratios of histopathologically validated cortical lesions to surrounding normal appearing grey matter and grey matter to white matter for the included MRI sequences are described in Table 3. Contrast ratios for cortical lesions to normal-appearing grey matter were 0.19 for conventionally acquired DIR, 0.03 for artificially generated DIR from 3D-T_1_ and PD/T_2_, 0.04 for artificially generated DIR from 3D-T_1_ and FLAIR, and 0.02 for 3D-T_1_. For cortical lesions to normal appearing grey matter contrast ratios, differences between sequences were statistically significant for conventional DIR vs. both variants of artificial DIR (*P* = 0.003 for DIR from T_1_ and FLAIR, and *P* = 0.006 for DIR from T_1_ and PD/T_2_) and for conventional DIR vs. 3D-T_1_ (*P* = 0.018). There were no further significant differences between sequences.

**Table 3:** Contrast ratios.

	Conventional DIR	DIR from 3D-T_1_ + PD/T_2_	DIR from 3D-T_1_ + FLAIR	3D-T_1_
CR CL-GM	0.19 (0.21)	0.03 (0.06)^a^	0.04 (0.14)^a^	0.02 (0.16)^a^
CR GM-WM	3.81 (1.24)	0.48 (0.33)^a,b^	0.58 (0.18)^a,b^	0.10 (0.37)^a^

Data are presented as contrast ratio (CR; ± SD); contrast ratio is defined as (SI_1_–SI_2_)/SI_2_. CL–GM = cortical lesion to grey matter; GM–WM = grey matter to white matter; ^a^statistically significant difference (i.e., *P* < 0.05) compared to conventional DIR, ^b^statistically significant difference compared to 3D-T_1_.

Contrast ratios for normal-appearing grey matter to white matter were 3.81 for conventionally acquired DIR, 0.48 for artificially generated DIR from 3D-T_1_ and PD/T_2_, 0.58 for artificially generated DIR from 3D-T_1_ and FLAIR, and 0.10 for 3D-T_1_. Grey matter to white matter contrast ratio differences were significant between conventional DIR and both variants of artificially generated DIR (*P* = 0.001 for T_1_-FLAIR DIR and *P* = 0.001 for T_1_-PD/T_2_ DIR) as well as conventional DIR vs. standard 3D-T_1_ (*P* = 0.001). Differences in grey matter to white matter contrast ratios were also significant for both variants of artificially generated DIR vs. standard 3D-T_1_ (*P* = 0.001 for T_1_-FLAIR DIR and *P* = 0.001 for T_1_-PD/T_2_ DIR).

### Common artefacts

**Figure 4:**
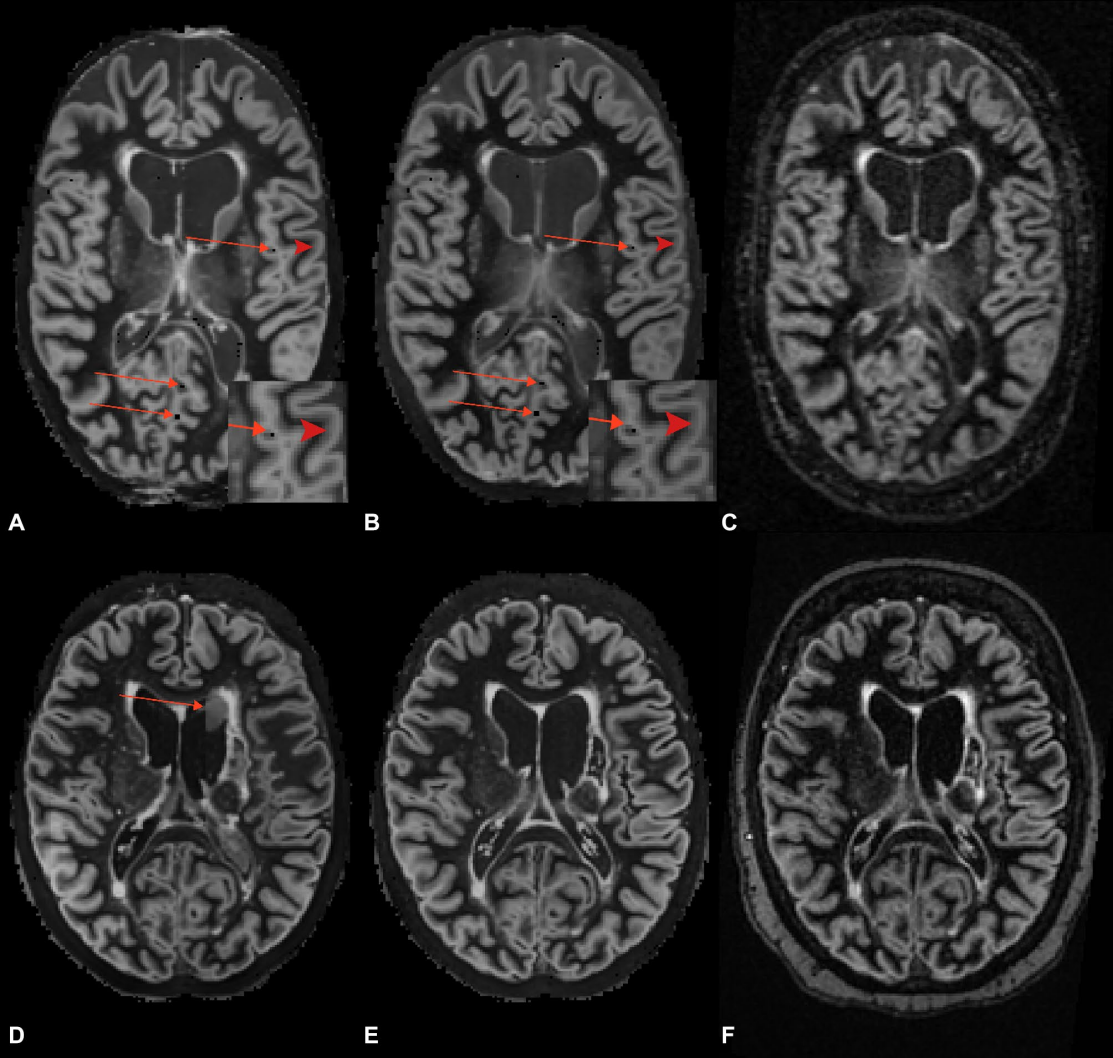
Artefacts in artificially generated images. (**A**) Artificial double inversion recovery (DIR) image generated from 3D-T_1_ and PD/T_2_ in which the arrows indicate ‘point artefacts’ and the chevrons indicate ‘rim artefacts’ in the cortex, which are also visible on artificial DIR images generated from 3D-T_1_ and fluid-attenuated inversion recovery (FLAIR). (**C**) Conventionally acquired counterpart of the artificially generated images in (**A**) and (**B**). (**D**) ‘Patch artefact’ in artificial DIR image generated from 3D-T_1_ and PD/T_2_, indicated by the arrows. (**E**) Corresponding artificial DIR generated from 3D-T_1_ and FLAIR and (**F**) corresponding conventionally acquired DIR (generated using Adobe Illustrator—Adobe Inc., 2019. Adobe Illustrator, Available at https://adobe.com/products/illustrator).

Although the artificially generated DIR images were less noisy than the conventionally acquired DIR images, some artefacts were detected in the data. Detected artefacts included ‘rim-artefacts’ (i.e. a hyperintense rim-forming in the cortex), ‘point-artefacts’ (i.e., black pixels throughout the image; potentially arising from zero-values in the 3D-T1 input images) and ‘patch-artefacts’ (i.e. subtle lines remaining from patch-wise augmentation of the images), which were detected in data from different MR systems and different ground contrasts. Rim-forming artefacts were visible in all artificial DIR images. Point-artefacts were detected in both variants of the artificially generated DIR images (3D-T_1_ and PD/T_2_ and 3D-T_1_ and 3D-FLAIR) which had their ground contrasts acquired on the GE MR system, whereas patch-artefacts were visible in artificially generated DIR from 3D-T_1_ and PD/T_2_ but not 3D-T_1_ and 3D-FLAIR from the Avanto system. In all artificially generated DIR images, the cortex showed constant but subtle hyperintensity, making it more difficult to distinguish between the different cortical layers. The artefacts that were noted are shown in Fig. 4.

## Discussion

Cortical multiple sclerosis lesions are better discernible on double inversion recovery images, but these are often unavailable. We have evaluated the sensitivity and histopathological specificity of artificially generated double inversion recovery images in clinically and pathologically confirmed patients with multiple sclerosis. With a combined postmortem in situ brain MRI and histopathology approach, we have found no differences in histopathology-blinded lesion detection between conventional DIR images and two variants of artificially generated DIR images. This implicates that artificially generated DIR images could substitute conventional DIR images when unavailable or in case of limited acquisition time.

Vis-à-vis comparison of histopathology-blinded and unblinded detection rates showed that a priori detection of cortical lesions could be improved on all sequences, although most on artificial DIR images generated from 3D-T_1_ and 3D-FLAIR. Differences between the two variants of artificially generated DIR images in cortical lesion detection potential could be attributed to the input contrasts, 2D-PD/T_2_ vs. 3D-FLAIR. The latter had a higher resolution and hence more potential to pick up small signal anomalies. Thus, with the current sequence parameters, artificial DIR images generated from 3D-T_1_ and 3D-FLAIR have the most potential for cortical lesion detection.

Artificially generated DIR images have been shown sensitive to cortical multiple sclerosis lesions, but none of these works included histopathological validation^10,11^. The relatively low sensitivity (i.e., < 25%) and high specificity (i.e., > 90%) of conventional DIR that were found are consistent with previous works^6,7^. Contrasting to the existing literature, no significant difference between conventional DIR and T_1_ was found in the number of lesions that were detected blinded to histopathology. However, previous works using histopathology as gold standard included 2D-T_1_-weighted images, while we included 3D-T_1_^6,7^. Nonetheless, we do see –concordant with former literature– 3D-T_1_-weighted images falling behind in terms of lower intra- and inter-rater scores. Furthermore, 3D-T_1_-weighted images have lower histopathological specificity compared to conventionally acquired and both variants of the artificially generated DIR images, implying that standard 3D-T_1_ weighted images are more difficult to assess for cortical lesions.

In both conventional and artificial DIR images, > 75% of cortical lesions is missed a priori—a recurring issue in MRI-histopathology studies^6–8,18,19^. Thus, detection potential of artificial DIR images does not surpass that of conventionally acquired DIR images. This could be due to low contrast ratios in the images, in combination with small cortical lesion size. Furthermore, like conventionally acquired DIR images, artificially generated DIR images are host to artefacts, albeit of a different nature. These artefacts did not affect the sensitivity and specificity of the artificially generated DIR images in the current sample. Further development of the algorithm (e.g. adjusting patch-wise augmentation component) should suffice to increase the contrast ratios and minimize or remove these artefacts, which might increase sensitivity and specificity of the artificially generated images.

The body of literature examining the clinical relevance of cortical lesion detection is consistently increasing. In a recent study, cortical lesions were found to be among the main correlates of disease progression and long-term invalidity^20^. A priori cortical lesion detection remains intricate, implicating that reader training could be improved or that the scoring criteria could be adjusted^16^. Another way to mitigate this detection issue would be through automated detection of lesions. However, performance of such detection tools has not yet approached manual lesion detection rates^21–23^. Other combinations of input image contrasts could also be used to increase cortical lesion detection: artificial DIR (or PSIR) images could be generated from e.g. T_2_* combined with MPRAGE sequences, since (combinations of) these sequences have been shown fruitful for cortical lesion detection several times before^6,24–27^.

Limitations to this work include that the 3D-T_1_ and 3D-FLAIR sequence had suboptimal nulling in some patients, due to changes in the brain after death^28^. Standardized inversion time, and nulling of 3D-T_1_ was suboptimal for some patients due to range in postmortem delay. Also, there were no control patients included to assess whether the algorithm might have shown cortical lesions in controls as well. However, former work with artificial DIR images that were generated using a similar algorithm included controls and did not show any lesions in the latter^10^. Moreover, given the retrospective character of this study we were limited to the use of coronal brain slices whilst—concordant with the literature- the images were assessed in axial fashion. However, translation from axial to coronal plane was automated using the cross-hair function embedded in FSL View in order to prevent mistakes in translation. The a priori sensitivity of cortical lesions was not hampered by this additional processing step. A strong characteristic of the current work is that the ground contrasts from which the artificial DIR images were generated were acquired on different MRI scanners, underlining the scanner-robustness of the algorithm whilst maintaining sensitivity for cortical lesions. Future works should also include multi-centre validation of artificially generated images, having both input data and readers from different centres.

In conclusion, artificial DIR images, particularly generated from a combination of conventional clinical 3D-T_1_ and 3D-FLAIR images, could potentially substitute conventional DIR images for cortical lesion detection in patients with multiple sclerosis. This offers opportunities when conventional DIR images are unavailable, but also for ex post facto addition to clinical trial cohorts in which DIR had not been included a priori. Cortical lesion sensitivity for both artificially generated and conventionally acquired DIR images could be increased, as our results show potential for improvement of the a priori detection rates.

## Materials and methods

### Patients and autopsy procedure

Post-mortem imaging data and tissue samples were retrospectively obtained through the standardized Amsterdam MS Center rapid autopsy protocol between 2012 and 2018^14,15^. The protocol included in situ MR brain imaging, followed by brain extraction from cranium. Two MR systems were used for scanning, a 1.5 T system and 3 T system. Details of the MR protocol, including 3D-T_1_, 2D-PD/T_2_, 3D-FLAIR and 3D-DIR sequences, are provided in the supplementary methods. After in situ imaging, the brain was cut into coronal slices, from which tissue samples were sectioned: in part following a standardized protocol and in part following discernible pathology.

### Standard protocol approvals, registrations and patient consents

Prior to death, all patients had registered with the Dutch Brain Bank, thereby giving informed consent for the use of their tissue and medical records for research purposes. Permission for the autopsy protocol was granted by the Amsterdam UMC institutional ethics review board, approval number 2009/148. Histopathology of 11/38 patients has been used for validation of different MR sequences before^6^. All procedures were performed in accordance with the applying guidelines and regulations.

### MR image pre-processing and generation of artificial MR images

In brief, MR image pre-processing was performed using the Insight Toolkit (ITK; https://itk.org) and functional MRI of the brain (FMRIB) Software Library (FSL version 5.0.4; http://fsl.fmrib.ox.ac.uk) and consisted of rigid co-registration with MNI standard space using FLIRT (part of FSL) and spline interpolation. An extensive description of the network is provided elsewhere^10^. In short, two variants of artificial DIR images were generated through training of a convolutional neural network to predict 3D-DIR images, either from a combination of 3D-T_1_ and 3D-FLAIR images or from a combination of 3D-T_1_ and 2D-PD/T_2_ images. The network architecture combined a competing generator and discriminator network: the generator was trained to produce artificial 3D-DIR images from either 3D-T_1_ and FLAIR or from 3D-T_1_ and PD/T_2_, whereas the discriminator was trained to discriminate between artificially generated DIR images and their conventionally acquired counterparts. The network was trained using input data from different vendors. A total of 200 epochs were trained, using a two-folded data structure in which the patients were randomly appointed to train and test set in a 2:1 ratio. An overview of the train and test sets is presented in Table S1. Furthermore, patch-wise data-augmentation was performed. Optimization of the algorithm and initial quality assessment was performed through visual inspection of the artificially generated images, compared to their conventionally acquired counterparts.

### Histopathology-blinded lesion detection

To minimize patient recognition by the reader, all images were randomly ordered and flipped prior to scoring. Cortical lesions were manually scored in Medical Image Processing, Analysis and Visualization software (MIPAV; version 10.0.0, Centre for Information Technology, National Institutes of Health, Bethesda, MD, USA). Cortical lesions were scored in axial fashion following consensus guidelines developed by the MAGNIMS group for both artificially generated and conventionally acquired DIR images: cortical lesions had to be hyperintense areas compared to the surrounding grey matter, of at least 3 mm^2^^16^. For every scored lesion, adjacent slices were assessed to prevent scoring artefacts, e.g. from cortical vessels that can followed through adjacent slices. Lesion scoring was performed by P.M.B. (3 years of experience in cortical lesion scoring). Histopathology-blinded and unblinded inter-rater agreement was evaluated with J.J.G.G. (> 15 years of experience) for a subset of five patients for all sequences. Furthermore, histopathology-blinded intra-rater agreement was determined for all sequences of a random subset of five patients.

### Histopathological staining and lesion identification

Brain tissue was stained for proteolipid protein (myelin; PLP)—the histopathological staining protocol is provided in the supplementary methods. After tissue staining and histopathology-blinded MRI scoring, histopathological lesions were identified as areas of complete absence of myelin (i.e., lack of PLP). Cortical lesions were also subdivided into four types based on their position in the cortex: mixed grey-white matter lesions (type I; *leukocortical lesions*), purely intracortical (type II; *intracortical lesions*), subpial (type III) or pan-cortical (type IV) lesions^17^.

### Matching and unblinded MRI scoring

After histopathology-blinded MRI scoring, tissue samples were matched to the conventionally acquired DIR images through manual identification of the corresponding MRI regions. The matching procedure is illustrated in Fig. 1. To assure that no lesions were missed due to thin slicing and curving of the tissue sample in the coronal plane, unblinded assessment was expanded to the slices adjacent to lesion location. Contrast ratios for the different sequences were calculated; detailed descriptions are provided in the supplementary material.

### Statistical analysis

Sensitivity of the included sequences for cortical lesion detection was determined by dividing the number of lesions detected in the histopathology-blinded (or unblinded scorings) by the number of lesions detected on histopathology, times 100%. Specificity of the different sequences was determined by dividing the number of histopathologically validated lesions by the tot number of lesions that were scored. Comparisons between sequences and sequences to histopathology were made using linear mixed models in SPSS 26.0 (SPSS, Chicago, Illinois), controlling for age, sex and postmortem delay at time of commencement of the in situ MRI. Results were corrected for multiple comparisons using Fischer’s Least Significant Difference test, after which *P*-values of ≤ 0.05 were considered statistically significant. Inter- and intra-rater agreement were expressed as intra-class correlation coefficient, two-way mixed model, absolute agreement. Differences between contrast ratios were assessed using Wilcoxon signed-rank tests.

## Supplementary Information

Supplementary Information.
