# Supplementary material for: Artificial double inversion recovery images can substitute conventionally acquired images: an MRI-histology study
Source: Sci Rep. 2022 Feb 16;12:2620. doi: 10.1038/s41598-022-06546-4 (PMC8850613; doi:10.1038/s41598-022-06546-4)
Supplement: Supplementary file 1 — Supplementary Information. [file 41598_2022_6546_MOESM1_ESM.docx]

**SUPPLEMENTAL MATERIAL**

**Magnetic resonance imaging**

Post-mortem *in situ* imaging was performed using two systems. The first system that was used was a 1.5 Tesla whole-body scanner (Avanto; Siemens Medical Solutions, Erlangen, Germany) with an eight-channel phased-array head coil (In Vivo, Orlando, Fl). The protocol included a 3D-T_1_ weighted magnetization-prepared rapid-gradient echo (MPRAGE; repetition time (TR) 2700 ms, echo time (TE) 4.66 ms, inversion time (TI) 950 ms, 8° flip angle, sagittal 1.3 mm slices, 1.21 x 1.21 mm^2^ in-plane resolution, acquisition time (TA) 4m 50s) sequence, an axial spin-echo 2D-PD/T_2_ (TR 5710 ms, TE 22/99 ms, axial 3.0 mm slices, 1.0 x 1.0 mm^2^ in-plane resolution, TA 3m 50s) sequence, a 3D-FLAIR (TR 6500 ms, TE 355 ms, TI 2200 ms, sagittal 1.3 mm slices, 1.21 x 1.21 mm^2^ in-plane resolution, TA 5m 14s) sequence, and a 3D-DIR (TR 6500 ms, TE 355 ms, TI 350/2350 ms, sagittal 1.3 mm slices, 1.21 x 1.21 mm^2^in-plane resolution, TA 9m23s) sequence.

The second system that was used was a 3 Tesla whole-body scanner (GE Signa HDxt, Milwaukee, WI, USA), with an eight-channel phased-array head coil. The protocol included a 3D-T­_1_ weighted fast-spoiled gradient echo (FSPGR; TR 6.66 ms, TE 2.93 ms, TI 450 ms, 15° flip angle, sagittal 1.0 mm slices, 1.0 x 1.0 mm^2^ in-plane resolution, TA 5m 7s) sequence, an axial dual-echo PD/T_2_ (TR 4246 ms, TE 20/112 ms, axial 3.0 mm slices, 1.0 x 1.0 mm^2^ in-plane resolution, TA 4m 41s) sequence, a 3D-FLAIR (TR 8000 ms, TE 125.9 ms, TI 2247 ms, sagittal 1.2 mm slices, 0.97 x 0.97 mm^2^ in-plane resolution, TA 5m 39s) sequence, and a 3D-DIR sequence (TR 8000 ms, TE 126.4 ms, TI 725/4500 ms, sagittal 1.2 mm slices, 0.97 x 0.97 mm^2^ in-plane resolution, TA 9m 40s) sequence.

**Histopathological staining**

Brain tissue was stained following the same protocol as described in Bouman, Steenwijk, Pouwels, Schoonheim, Barkhof, Jonkman and Geurts ^6^. In brief, tissue samples from standardized regions were formalin-fixed for 48 hours and paraffin embedded, supplemented by regions with macroscopically and MRI-visible pathology. From the FFPE samples 10 μm-thick sections were cut. Sections were heated in the steam cooker for 30 minutes, submerged in Tris-EDTA buffer (10 mM; pH 9.0) to perform antigen retrieval. Then, endogenous peroxidase was blocked using 1% hydrogen peroxide in tris buffer saline (TBS; pH 7.6). To block for non-specific binding, sections were incubated with 3% bovine serum albumin in TBS-tx. Next, sections were incubated with primary antibody proteolipid protein (PLP; Bio-Rad, Hercules, CA, US) overnight at 4°C. Subsequently, incubation with biotin labelled donkey-anti-mouse (DoaM; Jackson IgG, Cambridgeshire, UK) 1:400 diluted in TBS-tx for 2 hours, was performed. Then, sections were incubated with ABC (Vector, Burlingame, CA, US) diluted in 1:400 TBS-tx for 1 hour. Colour development was performed using 3’3’-Diaminobenzidine (DAB) for 10 minutes. Then, counterstaining with thionin (Brand, Wertheim, Germany) was performed.

**Calculation of contrast ratio**

For all included sequences (artificially generated DIR from T­_1_ and PD/T_2,_ and from T_1_ and FLAIR, conventionally acquired DIR, and T_1_), contrast ratios were calculated. Contrast ratios were calculated in a random subsample of five patients, based on signal intensity measures in different regions of interest; cortical lesions (N = 15), normal appearing grey matter (N = 15) and normal appearing white matter (N = 15). Contrast ratio was defined as (SI_1_ – SI_2_) / SI_2_, in which SI_1_ denotes signal intensity of the lesion or normal appearing grey matter and SI_2_ denotes signal intensity of the normal appearing grey matter or normal appearing white matter. Outcomes of all selected patients were then averaged to generate one contrast ratio measure for cortical lesions to normal-appearing grey matter and normal appearing grey matter to white matter for each sequence.

**Table S1 Overview of train and test sets**

|  | **Fold 1** | | **Fold 2** | |
| --- | --- | --- | --- | --- |
| **Vendor** | Train 1 | Test 1 | Train 2 | Test 2 |
| Sonata | 7 | 3 | 7 | 3 |
| GE | 17 | 8 | 16 | 9 |

*Note.* Patients are randomly appointed to the two folds.


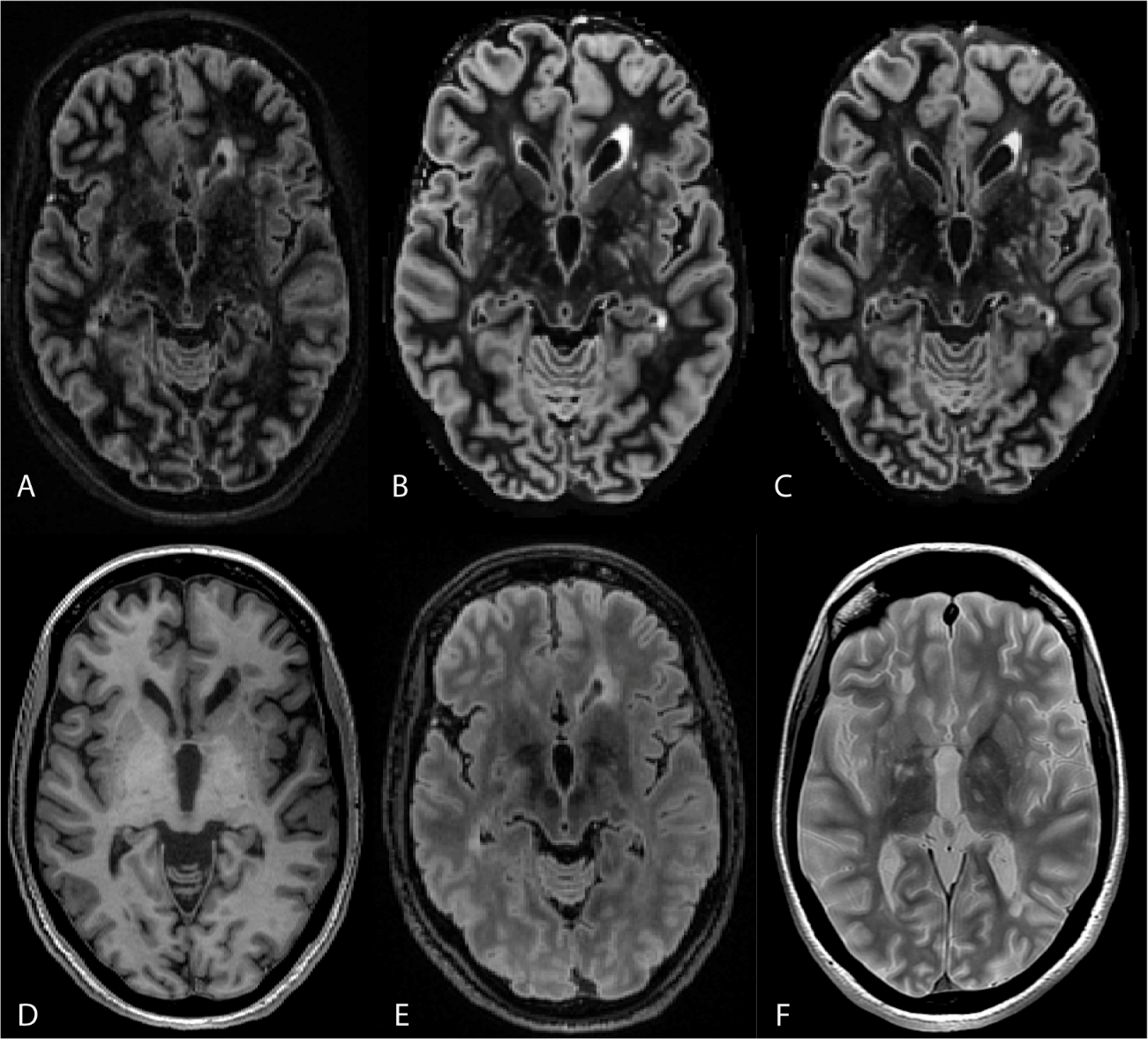


**Figure S1.** Overview of DIR sequences and their input sequences at 3 Tesla. (A) Conventionally acquired DIR, (B) Artificially generated DIR from combined 3D-T1 and 3D-FLAIR, (C) artificially generated DIR from combined 3D-T1 and 2D-PD/T2, (D) 3D-T1, (E) 3D-FLAIR, (F) 2D-PD/T2 (generated using Adobe Illustrator – Adobe Inc., 2019. Adobe Illustrator, Available at https://adobe.com/products/illustrator).


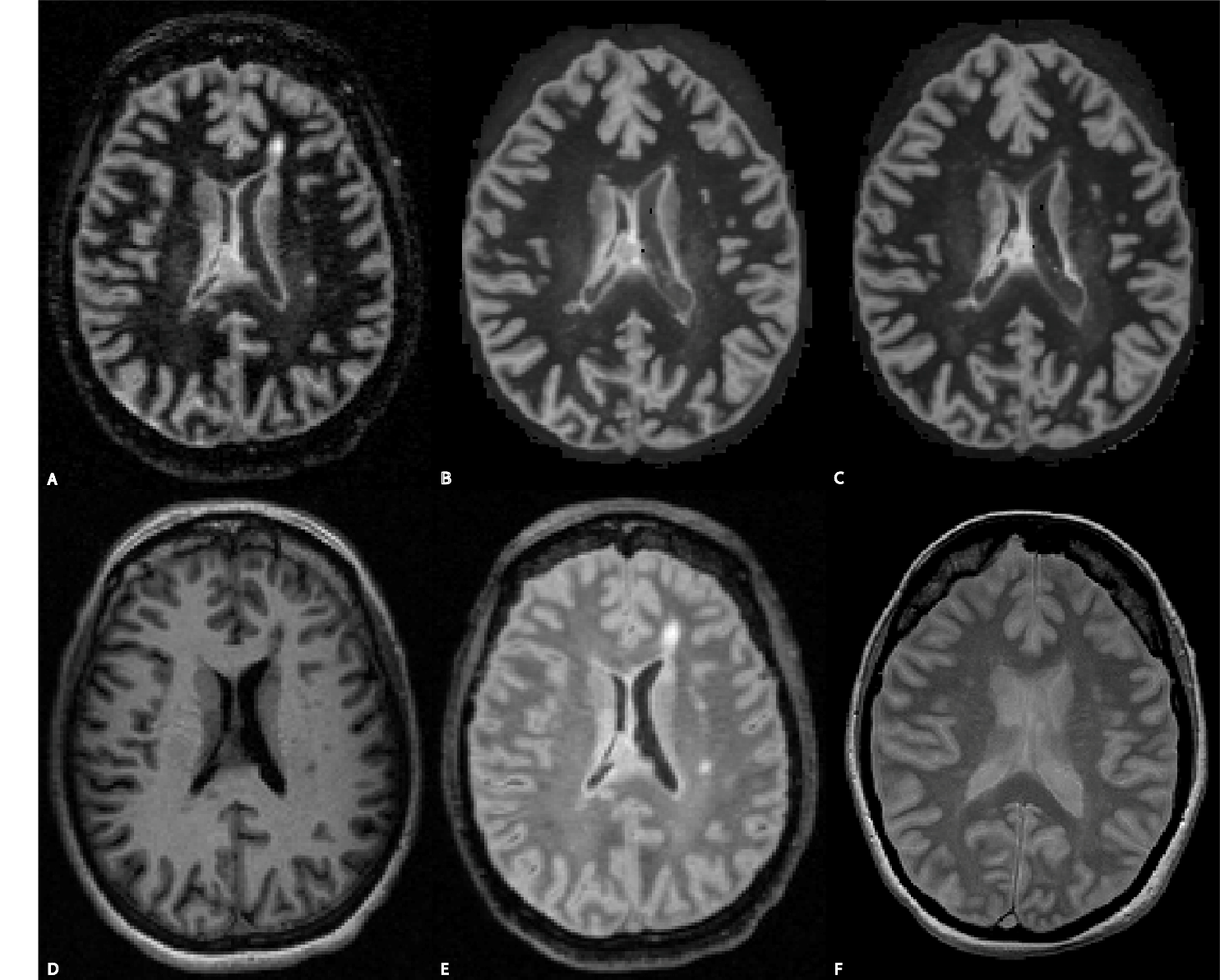


**Figure S2.** Overview of DIR sequences and their input sequences at 1.5 Tesla. (A) Conventionally acquired DIR, (B) Artificially generated DIR from combined 3D-T1 and 3D-FLAIR, (C) artificially generated DIR from combined 3D-T1 and 2D-PD/T2, (D) 3D-T1, (E) 3D-FLAIR, (F) 2D-PD/T2 (generated using Adobe Illustrator – Adobe Inc., 2019. Adobe Illustrator, Available at https://adobe.com/products/illustrator).
